# Local cryotherapy minimally impacts the metabolome and transcriptome of human skeletal muscle

**DOI:** 10.1038/s41598-017-02754-5

**Published:** 2017-05-25

**Authors:** Dylan C. Sarver, Kristoffer B. Sugg, Nathaniel P. Disser, Elizabeth R. Sibilsky Enselman, Tariq M. Awan, Christopher L. Mendias

**Affiliations:** 1 Department of Orthopaedic Surgery, University of Michigan Medical School, Ann Arbor, MI USA; 2 Department of Molecular & Integrative Physiology, University of Michigan Medical School, Ann Arbor, MI USA; 3 Department of Surgery, Section of Plastic Surgery, University of Michigan Medical School, Ann Arbor, MI USA

## Abstract

Cryotherapy is commonly used in the treatment of skeletal muscle injuries. However, the data to support the use of cryotherapy is inconclusive, and the biochemical etiology of cryotherapy in human skeletal muscle remains largely unknown. We therefore sought to determine how a clinically-relevant dose of cryotherapy would impact the transcriptome and metabolome of skeletal muscle. Eight healthy male subjects (age 24.7 ± 4.5 years, BMI 22.2 ± 1.6) received a 15 minute bout of local cryotherapy, delivered via ice cup massage over the anterolateral thigh. This resulted in an 85% decrease in skin temperature and a predicted 27% reduction in intramuscular temperature. The contralateral side served as a non-treated control. Two hours after cryotherapy, muscle biopsies were obtained to analyze changes in the transcriptome, metabolome, and activation of p38 MAPK, ERK1/2, Akt, and p70S6K proteins. No changes were detected in the transcriptome between control and cooled muscles. Cryotherapy reduced levels of hexose sugars and hypoxanthine by 1.3%, but no statistically different changes were observed in 60 additional metabolites. Overall, no differences in phosphorylated p38 MAPK, ERK1/2, Akt, and p70S6K were observed. A clinically relevant dose of cryotherapy produced negligible acute biochemical and molecular changes in the skeletal muscle of human subjects.

## Introduction

Skeletal muscle injuries are among the most prevalent types of injuries observed in the sports medicine setting^1^. Cryotherapy is commonly used to treat skeletal muscle injuries, and is thought to work by reducing inflammation, decreasing metabolic demands of tissue and reactive oxygen species production, and promoting the general regenerative response of tissues^1–3^. Cryotherapy is frequently administered in either a static form, such as an ice bag, for a period of 15 to 30 minutes, or in an active form, such as ice cup massage, for a period of 10 to 15 minutes^1–4^. These doses of cryotherapy typically result in a 3–8 °C decrease in intramuscular (IM) temperature that persist up to an hour or more after application^3, 5–7^, and are thought to alter levels of small molecule metabolites in tissue^8^. Despite the frequent use of topical cryotherapy, there is inconclusive evidence to support its use in the treatment of skeletal muscle injuries, and the precise biological mechanisms of action of cryotherapy are not well understood^9, 10^.

Numerous animal and *in vitro* cell culture studies have evaluated the therapeutic use of cold in experimental models of muscle injury and regeneration, and have reported mixed results of cryotherapy on outcomes associated with improved muscle regeneration^11–15^. Two limiting factors in many of these studies are that the extent of cooling is either much greater than what is typically used clinically, or that the cooling is administered via immersion of large portions of the body, resulting in alterations in core temperature and potential systemic effects that are not observed in local treatments. Numerous studies across different types of organisms and tissues have demonstrated that prolonged exposure to cold is also known to induce the expression of so-called “cold shock” genes, CIRBP, CSDE1, RBM3 and YBX1, which encode proteins important for RNA processing and stability^16^. These cold shock genes are also upregulated during skeletal muscle hypertrophy and may play a role in promoting muscle growth and protection of muscle fiber nuclei against apoptosis^17, 18^, but it was unknown whether these genes are impacted by a clinically relevant dose of cryotherapy.

As the evidence to support the use of cryotherapy is inconclusive, and the biological effects of topical cooling have not been well studied in humans, we sought to conduct a basic science study to better define the physiological effects of cryotherapy in human muscle. To accomplish this, we measured changes in the transcriptome, metabolome, and in the activation of signaling pathways involved in muscle growth following a single bout of cryotherapy. Based on the proposed mechanisms of action of cryotherapy and previous studies in animal models, and the involvement of cold shock genes in muscle hypertrophy, we hypothesized that a single therapeutic application of cold would result in wide-spread changes in the metabolome and transcriptome of muscle tissue, and activate the Akt and p70S6K signaling pathways. To test this hypothesis, we used a paired design in healthy subjects where we administered a clinically relevant dose of cryotherapy to one leg, while the other leg served as a control. Two hours after cryotherapy, biopsies were obtained from the treated and untreated muscles, and subjected to biochemical and molecular analyses.

## Results

**Figure 1:**
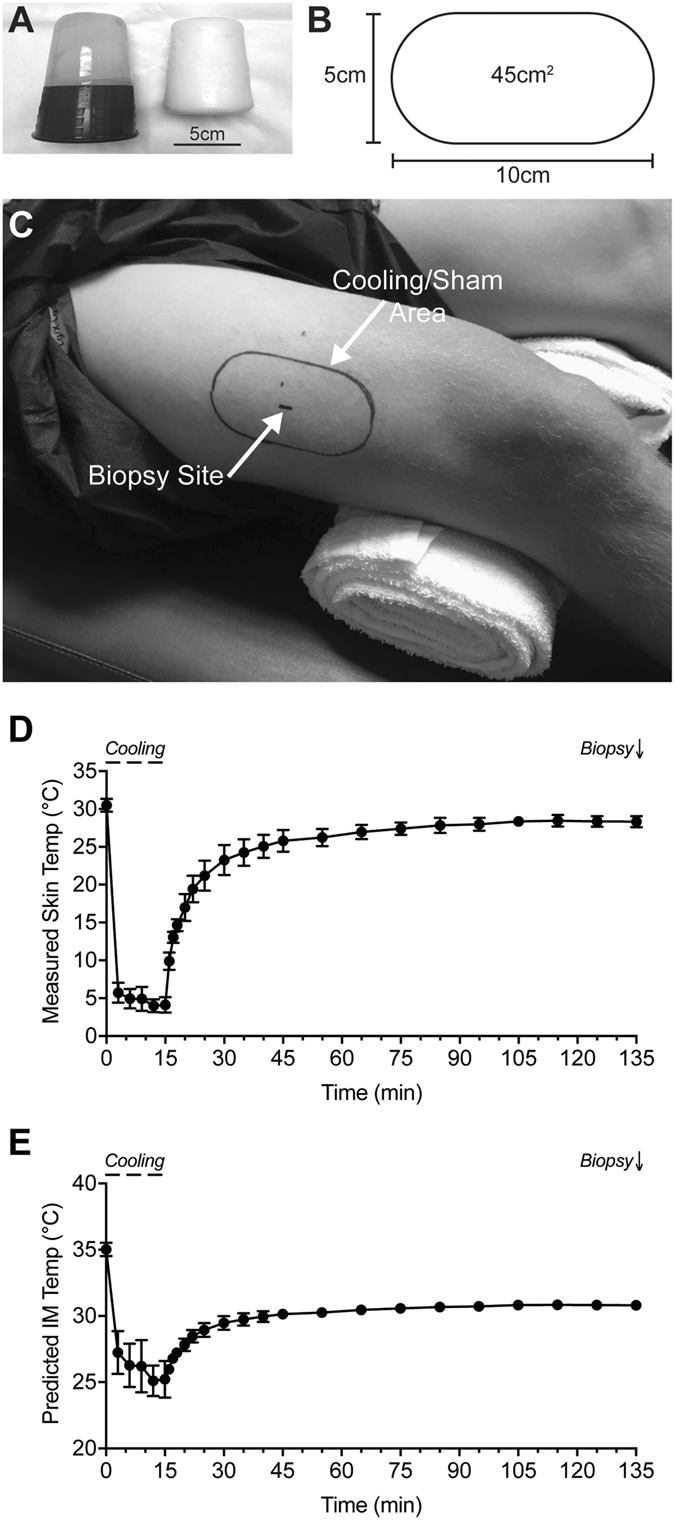
Overview of treatment area and temperature measurements. (**A**) Ice cup (left) and sham mold (right). (**B**) Template used to create an outline of the area to apply the cryocup or sham mold. (**C**) Demonstration of the area of the template and biopsy location on a subject. (**D**) Measured skin temperature values and (**E**) predicted intramuscular (IM) temperature measurements. Values are mean ± SD.

Results are presented as mean ± SD. The age of subjects in the study was 24.7 ± 4.5 years, BMI was 22.2 ± 1.6, and Tegner physical activity score was 7.3 ± 1.2. An overview of the ice cup mold, sham mold, and treatment size and area are presented in Fig. 1A–C. Using ultrasound, subjects had a subcutaneous fat thickness of 3.5 ± 2.8 mm in the region of biopsy. The measured skin temperature values are shown in Fig. 1D, and the predicted IM temperatures are shown in Fig. 1E. From the start to the end of the cooling, skin temperature decreased by 85%, resulting in a 27% predicted decrease in muscle temperature. By the time of biopsy, skin temperature remained 4% colder than the starting value, and the predicted IM temperature was 12% lower. Core temperature was 36.3 ± 0.2 °C, and for all subjects varied less than 0.2 °C throughout the study, indicating that local cryotherapy did not change core temperature.

**Figure 2:**
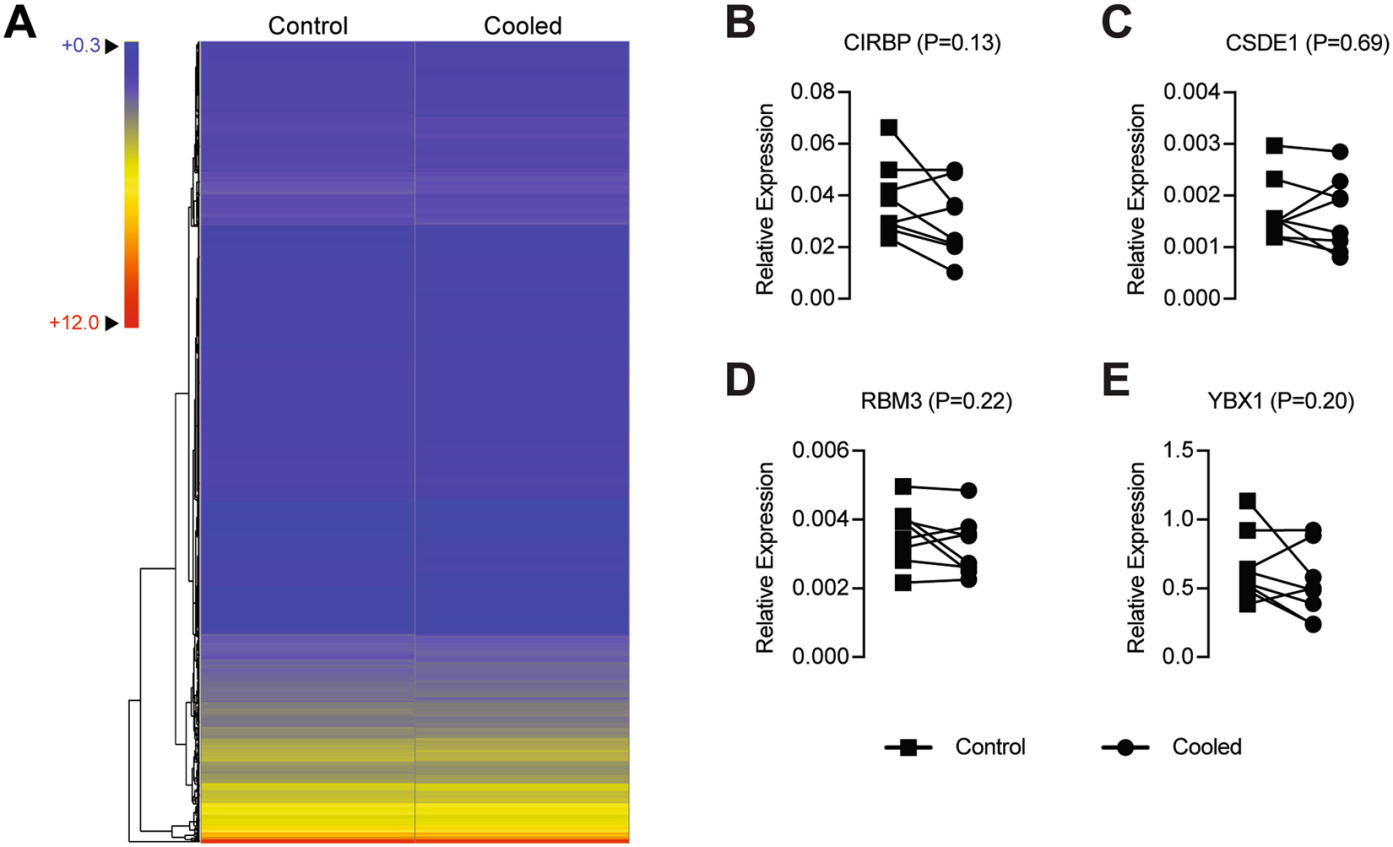
Heat map and gene expression. (**A**) Heatmap of microarray data from control and cooled muscles. Targeted gene expression measurements of the cold shock genes, (**B**) CIRBP, (**C**) CSDE1, (**D**) RBM3 and (**E**) YBX1, from control and cooled muscles. The expression of each gene was normalized to the stable housekeeping gene B2M. Differences were tested using paired t-tests, and p-values are indicated in each panel. N = 8 subjects.

For gene expression measurements, microarray analysis failed to identify any genes that were significantly different from each other. This is also shown in the heat map of the microarray data (Fig. 2A). While no differences were detected using microarrays, using qPCR we sought to measure changes in the cold shock genes, CIRBP, CSDE1, RBM3, and YBX1, which are reported to be induced after prolonged exposure to low temperatures. No differences were detected in relative levels of cold shock genes (Fig. 2B–E).

**Figure 3:**
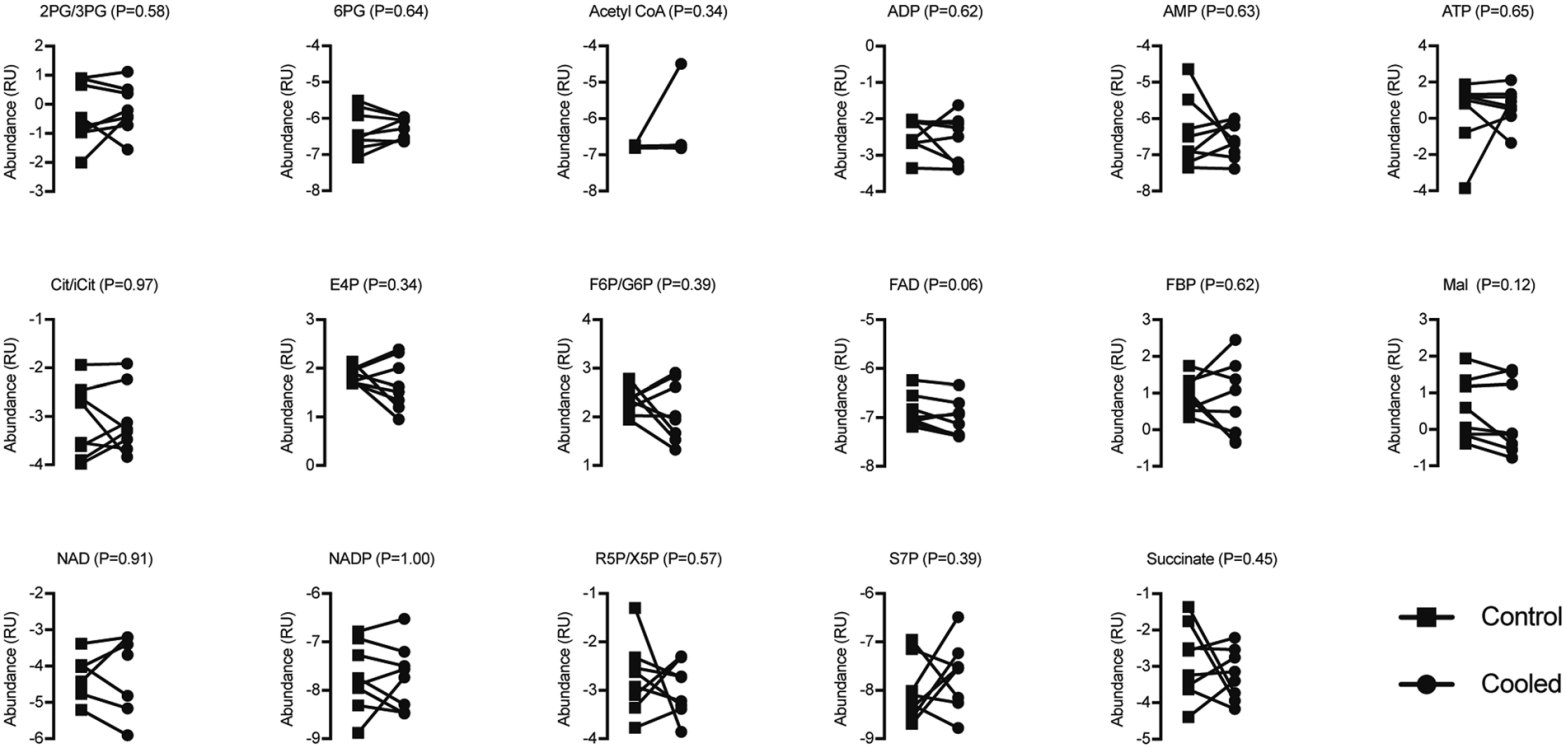
Metabolites with available isotopic standards. Relative abundance of 17 analytes measured in control and cooled muscles. Data were log_2_ transformed prior to analysis. Differences were tested using paired t-tests, and p-values are indicated in each panel. N = 8 subjects.

**Figure 4:**
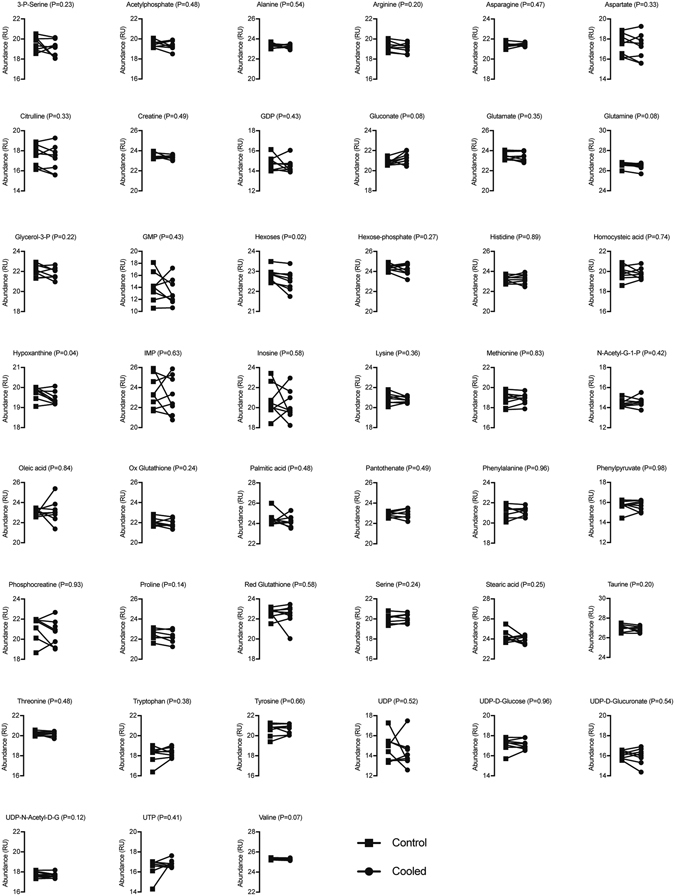
Metabolites without available isotopic standards. Relative abundance of 45 analytes measured in control and cooled muscles. Data were log_2_ transformed prior to analysis. Differences were tested using paired t-tests, and p-values are indicated in each panel. Only hexoses and hypoxanthine were significantly different between control and cooled samples. N = 8 subjects.

We then measured differences in metabolites involved in glycolytic, oxidative and amino acid metabolism (Figs 3 and 4). Of the 62 analytes measured, only hexoses (six carbon monosaccharides, which include glucose, galactose and fructose) and hypoxanthine were significantly different, both of which displayed an approximate 1.3% decrease in cooled tissue compared to controls (Fig. 4).

**Figure 5:**
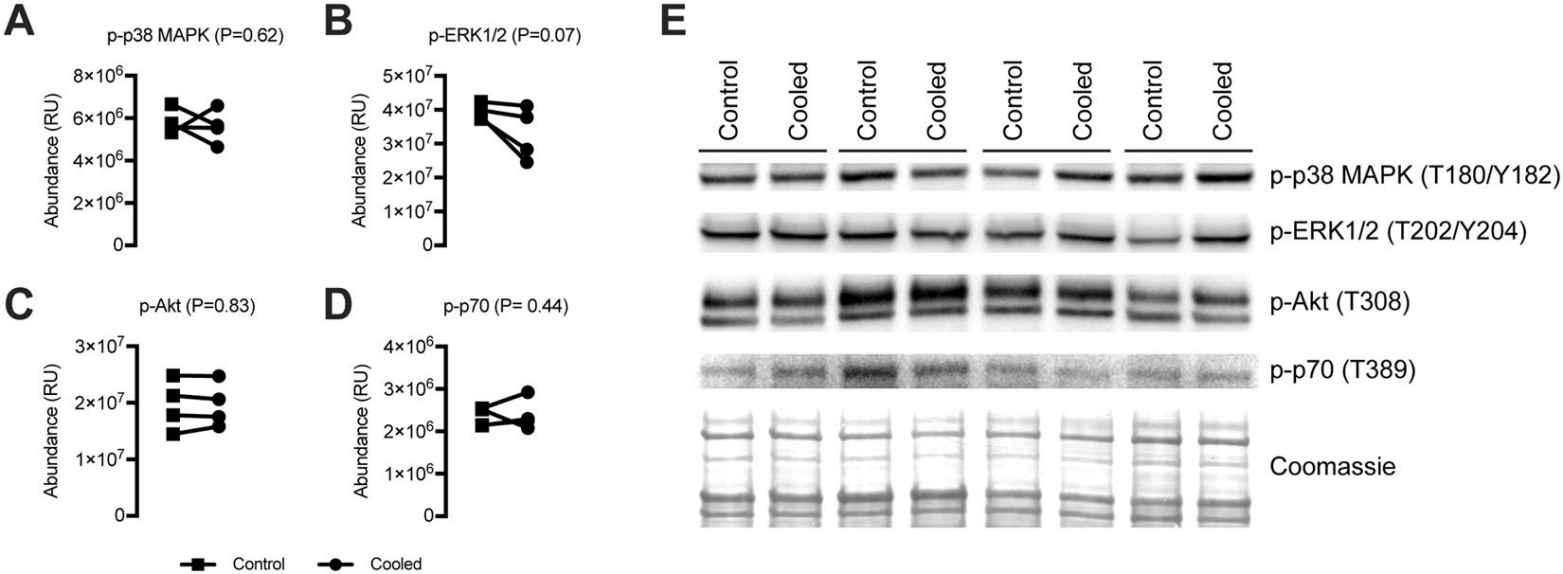
Immunoblots. Quantitative band densitometry analysis of immunoblots for (**A**) p-p38 MAPK, (**B**) p-ERK1/2, (**C**) p-Akt and (**D**) p-p70S6K from control and cooled muscles. Differences were tested using paired t-tests, and p-values are indicated in each panel. (**E**) Actual immunoblots of analyzed tissue. A Coomassie stained membrane is shown as a control for protein loading. N = 4 subjects.

Finally, we measured changes in phosphorylation of proteins important in metabolism, protein synthesis, and mechanotransduction in muscle. No differences in p38 MAPK, ERK1/2, Akt, or p70S6K phosphorylation were observed between control and cooled muscles (Fig. 5A–E).

## Discussion

Cryotherapy is among the most common therapeutic modalities used to treat skeletal muscle injuries, but there is a lack of biological and epidemiological evidence to support its widespread use^1, 3, 10^. We hypothesized that a single therapeutic application of cold would result in wide-spread changes in the metabolome and transcriptome of muscle tissue. However, to our surprise, we found cryotherapy induced very few changes in the metabolome, and no changes in the transcriptome or in the activation of p38 MAPK, ERK1/2, Akt, or p70S6K in muscle. The combined results from this work indicate that a single dose of cryotherapy, administered following standard of care guidelines, has a negligible acute impact on gene expression, cellular metabolism, and signal transduction pathways important in muscle growth and metabolism.

There are generally two different types of cryotherapy used in sports medicine. Local cryotherapy is where ice or cold packs are administered to a specific region of a limb or defined area on the trunk or head. This type of cryotherapy, which is typically used to treat a local injury, changes the temperature of the tissue in the immediate area but does not change core temperature^5, 19^. Less common than topical use, cryotherapy can also be administered systemically to large portions of the body, either through immersing an entire limb or large region of the body in cold water, or exposing the area to extremely cold air. For this second type of cryotherapy, which is often used in an attempt to enhance recovery following a bout of exercise or improve training and conditioning, IM temperature can decrease by 6 °C and core temperature up to 2 °C^19, 20^. As core temperature is impacted by systemically administered cryotherapy, and changes in circulating levels of catecholamines and cytokines are observed in this form of cryotherapy^20, 21^, the mechanism of action between local and systemic cryotherapy are likely different. As such, we will largely focus our discussion on work evaluating local cryotherapy.

Previous studies have indicated that local cryotherapy, achieved through the application of an ice bag for 30 minutes to the anterior thigh, can result in a reduction of skin temperature by 80% and IM temperature, measured 2 cm deep to the subcutaneous fat layer, by 23%^5, 22^. Two hours after the delivery of cryotherapy in these studies, IM temperature still had not returned to pre-treatment levels. Several other studies have determined that topical cryotherapy across different anatomical sites can cool the temperature of muscle at the level which we obtained biopsies^3, 5–7, 22^. Although we did not directly measure IM temperature in this study, the skin temperature measurements and predicted IM measurements are consistent with the findings of Jutte and Merrick^5, 22^. Additionally, to ensure more uniform and rapid cooling of tissue, we used an ice cup approach instead of ice bags, as a previous study demonstrated ice cup therapy was 37% more effective than ice bags in reducing IM temperature in the gastrocnemius muscle^23^.

Much of our understanding of the basic biology of cryotherapy in skeletal muscle physiology comes from *in vitro* or animal studies. Decreasing the temperature of cultured muscle stem cells by 5 °C over normal culture conditions reduced their proliferation rate, but also decreased apoptosis^13^. When rats were subjected to an ischemia/reperfusion muscle injury, three hours of cryotherapy applied after the injury reduced neutrophil accumulation and reactive oxygen species formation, and enhanced the metabolic function of mitochondria^14^. In rats, following cryolesion-injury, repeated bouts of topical cryotherapy resulted in reduction of markers of reactive oxygen species formation^24^. In a rat muscle crush-injury model, however, 20 minutes of ice applied immediately after injury resulted in reduced inflammation in the acute phase, but in the long term delayed muscle regeneration^15^. Six hours of topical cryotherapy improved microcirculation and reduced immune cell infiltration after a contusion injury in rats^11^. Although these studies have provided useful information regarding mechanism, the dose and extent of cryotherapy is generally greater than what is typically used clinically^2^, and rodent muscle displays different metabolic properties and responses to environmental stimuli than human skeletal muscle^25^, which is why we sought to explore a clinically relevant dose of cryotherapy in human subjects.

For human skeletal muscle, locally reducing temperature generally reduces the ability of muscle to generate active tension^26, 27^, but little is known about how local tissue cooling affects biochemical and molecular processes in human muscle tissue^28^. One published model predicts a decrease of 0.17 mM of ATP and 0.54 mM of phosphocreatine per 1 °C reduction in IM temperature^8^, however local cooling of the quadriceps muscle group with a cuff perfused with 0 °C water for 1 hour does not change the levels of ATP, creatine or phosphocreatine^29^. Topical cooling also did not change ATP or phosphocreatine levels in hand muscles^30^. These experimental findings are consistent with the observed results in the current manuscript. Although the mechanism is not understood, hypoxanthine is released from cultured human neuronal progenitor cells in response to a hypothermic challenge^31^, and this might explain why hypoxanthine levels were lower in cooled muscles. Increased glucose oxidation helps to protect cultured cardiomyocytes from hypothermia-induced apoptosis^32^, and elevated glucose oxidation in response to cryotherapy may explain the observed decrease in hexose levels observed in the current study. In a wrist flexor eccentric injury study, topical cryotherapy increased the circulating levels of markers of muscle fiber damage, creatine kinase and myoglobin, but decreased the levels of pro-inflammatory cytokines, IL-6, IL-12, and TNF-α^33^. No genes related to inflammation were differentially regulated in this study. We are unaware of any studies which have looked at changes in the transcriptome of skeletal muscle in the context of cryotherapy, but lowering incubator temperature from 37 °C to 33 °C for 2 hours changed less than 1% of the transcriptome of cultured human umbilical endothelial cells^34^.

Cold shock genes and proteins are thought to play a role in muscle hypertrophy^17, 18^, and because of this, we sought to evaluate if cryotherapy induced the activation of signaling pathways involved in muscle growth. In terms of modulating signaling pathways, chronic cooling of human lung fibroblasts by adjusting the culture temperature from 37 °C to 25 °C did not impact Akt phosphorylation, but did reduce ERK1/2 phosphorylation by 50% or more^35^. Cooling also increased the levels of the cold shock proteins CIRBP and RBM3 in these cells by 2-fold or greater^35^. These cold shock proteins are also induced during skeletal muscle hypertrophy^17, 18^, but were not affected by cryotherapy in the current study. Overall, while there is a paucity of data regarding the therapeutic use of ice on human muscle *in vivo*, our findings are generally consistent with what data is available from the literature, and suggest that topical cryotherapy does not have a profound impact on the biochemistry or molecular biology of skeletal muscle.

There are several limitations to this study. We only used a single dose of cryotherapy and measured changes at one time point. We think that two hours is an appropriate time window, as other perturbations such as exercise can induce widespread transcriptional changes to skeletal muscle within two hours^36^. We evaluated the transcriptome and phosphorylation of selected proteins, but we did not directly measure changes in total protein abundance. We also selected p38 MAPK, ERK1/2, Akt and p70S6K as signaling proteins to evaluate based on their role in regulating numerous physiological processes in skeletal muscle^37^ and the correlation between cold shock genes and muscle hypertrophy^16^, but it is possible cryotherapy affects the activation of other signaling pathways that were not measured. We did not directly assess IM temperature for reasons discussed above, but substantial changes in skin temperature were observed in response to ice cup treatment, and several studies have shown that local cryotherapy is able to cool muscle by 3–8 °C at a depth of 2 cm^3, 5–7^. Prior to evaluating effects in injured skeletal muscle, we sought to determine how cryotherapy impacted otherwise healthy muscle. It is possible that cryotherapy could have a more profound effect on the biological processes of injured muscles. Despite these limitations, we think that this study provided an important contribution to our understanding of the mechanism of action of the therapeutic use of ice in humans.

Cryotherapy is a staple in the treatment of skeletal muscle and other soft tissue injuries^1^. Despite the widespread use of this modality, there is a surprising lack of evidence to support its use. This is the first work, to our knowledge, that provided a comprehensive biological evaluation of the acute effects of cryotherapy on the skeletal muscle. We observed that a clinically utilized dose of cryotherapy does not have a substantial impact on the transcriptome or metabolome of healthy muscle tissue. This largely agrees with epidemiological studies and meta-analyses which have failed to demonstrate a positive impact of cryotherapy on the treatment of skeletal muscle injuries^9, 10^. Given the high rates of skeletal muscle injuries in the physically active population, further work which explores the effect of cryotherapy on the cellular and molecular processes that regulate muscle repair after injury in humans is necessary to further refine the therapeutic use of cold in the sports medicine setting.

## Methods

### Abbreviations

A list of abbreviations is provided in Table 1.

**Table 1:** Abbreviations used in this study.

2PG/3PG	2-Phosphoglycerate/3-Phosphoglycerate
3-P-Serine	3-Phospho-Serine
6PG	6-Phosphogluconate
A_260_	Absorbance at 260 nm
A_280_	Absorbance at 280 nm
ADP	Adenosine Diphosphate
AMP	Adenosine Monophosphate
ATP	Adenosine Triphosphate
B2M	β2-Microglobulin
Cit/iCit	Citrate/Isocitrate
CRIBP	Cold Inducible RNA Binding Protein
CSDE1	Cold Shock Domain Containing E1
E4P	Erythrose 4-Phosphate
F6P/G6P	Fructose-6-Phosphate/Glucose-6-Phosphate
FAD	Flavin Adenine Dinucleotide
FBP	Fructose-Bisphosphate
GDP	Guanosine Diphosphate
Glycerol-3-P	Glycerol-3-Phosphate
GMP	Guanosine Monophosphate
Hexose-P	Hexose-Phosphate
IM	Intramuscular
IMP	Inosine 5′-Monophosphate
Mal	Malate
N-Acetyl-G-1-P	N-Acetyl-Glucosamine-1-Phosphate
NAD	Nicotinamide Adenine Dinucleotide
NADP	Nicotinamide Adenine Dinucleotide Phosphate
Ox Glutathione	Oxidized Glutathione
R5P/X5 P	Ribulose 5-Phosphate/Xylulose 5-Phosphate
RBM3	RNA Binding Motif Protein 3
Red Glutathione	Reduced Glutathione
S7P	Sedoheptulose 7-Phosphate
Suc	Succinate
UDP	Uridine 5′-Diphosphate
UDP-D-Glucose	Uridine 5′-Diphosphate-D-Glucose
UDP-D-Gluconate	Uridine 5′-Diphosphate-D-Gluconate
UDP-N-Acetyl-D-G	Uridine 5′-Diphosphate-N-Acetyl-D-Glucosamine
UTP	Uridine 5′-Triphosphate
YBX1	Y-box Binding Protein 1

### Human Subjects

This study was approved by the University of Michigan Medical School IRB (HUM00114172) and conformed to the Declaration of Helsinki. Informed written consent was obtained from subjects prior to participation in the study. Physically active subjects who were 18–40 years of age were eligible for inclusion. Subjects were excluded if they use tobacco products, have a history of cold intolerance or urticaria, lower extremity injury, or any major medical illness, disease, myopathy, or rheumatism. A total of 8 subjects were recruited, all male.

### Study Design

Subjects reported to the lab in the morning following an overnight fast, and were instructed to avoid any physical activity other than activities of daily living for 48 hours prior to testing. The height and weight of subjects were measured, and subjects then completed the Tegner physical activity survey^38^. Each leg of the subject was randomized to either undergo cryotherapy or serve as the sham control, so that each subject acted as their own control. A template was placed on the anterolateral thigh of the subject, and a surgical pen was used to outline the area of cooling or sham treatment. An ultrasound system (Logiq Book XP, GE Healthcare, Chicago, IL) was used to measure subcutaneous fat thickness in the outlined area, and plan for the muscle biopsy procedure.

After resting comfortably on a treatment table for 20 minutes, cryotherapy was administered to one leg through the use of a 5 cm^2^ reusable ice cup mold (Cryocup, Cryo Therapy, Monticello, MN). The ice cup mold had been filled with tap water and frozen overnight in a −20 °C freezer. The area of cryotherapy, as outlined by the template, was 45 cm^2^ and designed to accommodate two-widths of a 5 cm^2^ ice cup. The ice cup was swept from one end of the template to the other over a one second interval. The total time of delivery was 15 minutes. Skin temperature was measured prior to beginning cryotherapy, and every three minutes after beginning cryotherapy using an infrared dual laser thermometer (model eT650D, ennoLogic, Eugene, OR). After administering cryotherapy, temperature measurements were performed at 1 to 5 minute intervals for the next two hours. The contralateral limb was treated for 15 minutes with a sham poly-(methyl methacrylate) mold that was the same size as the ice cup. The sham mold was maintained at room temperature (24 °C), and delivered under the same conditions as the ice cup. Pilot studies determined that the sham mold did not induce a change in the temperature of skin, and surface temperature measurements therefore did not need to be performed at regular intervals on the sham side. Core temperature measurements were obtained from a Thermoscan5 tympanic membrane thermometer (Braun, Cincinnati, OH) prior to beginning cryotherapy, at the end of cryotherapy, and again at the time of biopsy.

Two hours following the administration of the ice cup or the sham therapy, a biopsy was obtained from the vastus lateralis muscle in the middle of the treatment area. The skin overlying the area was scrubbed with ChloraPrep (CareFusion, San Diego, CA) and the biopsy area was infiltrated with 3–5 mL of 1% lidocaine into the subcutaneous tissue. A 1 cm incision was made in the skin and fascia using a scalpel blade, and using ultrasound guidance a 4 g UCH muscle biopsy needle (Dixons Surgical Instruments, Wickford, UK) was placed into the muscle such that the cutting window of the needle was 2 cm deep to the subcutaneous fascia. Suction was applied, and the needle was passed to obtain a biopsy. This was repeated three times to obtain three biopsy samples. In order to be used for biochemical or molecular analyses, biopsies had to be at least 50 mg in mass. This resulted in complete sets of biopsies for all 8 subjects for metabolomics and gene expression measurements, and 4 for protein measurements. Biopsies were rapidly cleaned, weighed, snap frozen in liquid nitrogen, and stored at −80 °C. After the biopsy, the skin was closed using Dermabond (J&J, New Brunswick, NJ) reinforced with Steri-Strips (3 M, Saint Paul, MN).

### Estimation of Intramuscular Temperature

The muscle fibers of the vastus lateralis are relatively long, approximately 8 cm in length^39^, and to ensure that the placement of a temperature probe into the muscle did not impact observed changes in the transcriptome or metabolome of muscle, we relied on surface temperature measurements to predict IM temperature values. Skin temperature measurements were obtained as described above. We then used this data to estimate IM temperature changes based on the measurements of Jutte and colleagues^5^, who measured skin temperature and IM temperature 2 cm deep to the subcutaneous fat layer of the anterolateral thigh of subjects who were receiving ice bag cryotherapy.

### Gene Expression Measurements

RNA was isolated from muscle biopsies and gene expression was performed as previously described^40, 41^. Biopsies were homogenized in QIAzol (Qiagen, Valencia, CA), and RNA was isolated using a miRNeasy kit (Qiagen) supplemented with the use of DNase I (Qiagen). RNA quality was assessed using a BioAnalyzer system (Agilent, Santa Clara, CA) All samples had A_260_/A_280_ ratios greater than 1.8 and RNA integrity numbers greater than 8.0. After reverse transcription of 200 ng of RNA using iScript supermix (Bio-Rad, Hercules, CA), quantitative PCR (qPCR) was conducted in a CFX96 real time thermal cycler using iTaq SYBR green supermix reagents (Bio-Rad). A list of RNA transcripts and primer sequences is provided in Supplementary Table S1. The 2^−ΔCt^ technique was used to normalize the expression of mRNA transcripts to the stable housekeeping gene β2-microglobulin (B2M), and differences between cooled and sham muscles were tested using paired t-tests (α = 0.05) in Prism version 7.0 (GraphPad Software, La Jolla, CA).

Microarray measurements were performed by the University of Michigan DNA Sequencing Core following manufacturer recommendations as previously described^40^. A total of 100 ng of RNA from cooled and control muscle biopsies of 6 subjects were analyzed. RNA was prepared for microarray analysis using a Pico WTA system (NuGen, San Carlos, CA) and hybridized to Human Gene ST 2.1 strips (Affymetrix). Expression values were calculated using a robust multi-array average, and data were log_2_ transformed. Differences between control and cooled samples were calculated using paired t-tests (α = 0.05), and p-values were adjusted for multiple comparisons using a Benjamini-Hochberg false discovery rate of 0.05. ArrayStar version 13 (DNASTAR, Madison, WI) was used generate a heat map using hierarchical clustering with Euclidean clustering and centroid linkage. The microarray dataset has been uploaded to the NIH GEO database (accession ID GSE89097).

### Metabolomics

Metabolomics measurements were performed by the University of Michigan Metabolomics Core as previously described^42^. Muscle biopsies were homogenized and metabolites extracted using an 8:1:1 mixture of methanol, chloroform and water containing isotope-labeled internal standards. Liquid chromatography-mass spectrometry analysis was performed in an Agilent system consisting of a 1260 UPLC module coupled with a 6520 quadrupole-time-of-flight mass spectrometer. Metabolites were separated using a 150 × 1 mm Luna NH_2_ hydrophillic interaction chromatography column (Phenomenex, Torrance, CA). The mass spectrometer was operated in electrospray ionization mode, and data were processed using MassHunter software (Agilent). Metabolites were either normalized to the nearest isotope-labeled internal standard and quantitated using two replicated injections of five standards to create a linear calibration curve with accuracy better than 80% for each standard, or normalized to the nearest internal standard, and the peak areas were used for differential analysis. MetaboAnalyst 3.0 software (McGill University, Montreal, QC) was then used to log_2_-transform data and perform paired t-tests (α = 0.05) on the analyzed data. Because isotopic standards were available for some but not all of the metabolites, the data were log_2_ transformed and analyzed separately between the two groups, and presented in two figures.

### Immunoblots

Immunoblots were performed as previously described^40, 43^. Muscle biopsies were homogenized in ice cold RIPA Lysis and Extraction Buffer (ThermoFisher Scientific, Grand Island, NY) supplemented with a 1:100 protease and phosphatase inhibitor cocktail (ThermoFisher Scientific). After homogenization and sonication, lysates were spun at 13,000 × g, and the supernatants were collected. Protein concentration of samples was measured using a BCA Protein Assay Kit (ThermoFisher Scientific). Samples were then diluted in Laemmli sample buffer (Bio-Rad), placed in boiling water for 2 minutes, and 20 µg of protein was separated on AnyKD gels (Bio-Rad). Proteins were transferred to either nitrocellulose (p38 MAPK and Akt) or PVDF (ERK1/2 and p70S6K) membranes using the Trans-Blot SD semi-dry transfer apparatus (Bio-Rad), blocked with 5% bovine serum albumin, and incubated with rabbit primary antibodies (1:1000) from Cell Signaling Technology (Danvers, MA) against phosphorylated Akt (T308, catalog # 13808), phosphorylated ERK1/2 (T202/Y204, catalog # 4511), phosphorylated p38 MAPK (T180/Y182, catalog # 9211), phosphorylated p70S6K (T389, catalog # 9430). Primary antibodies were detected using a goat anti-rabbit HRPO conjugated antibody (catalog # 7074, Cell Signaling Technology) at a concentration of 1:10000, along with Clarity enhanced chemiluminescent detection reagents (Bio-Rad). Membranes were imaged using a ChemiDoc imaging system (Bio-Rad), and band densitometry was calculated in relative units (RU). Following detection, membranes were stained with Coomassie Brilliant Blue to verify equal protein loading.

## Electronic supplementary material

Supplementary Table S1
