# Supplementary material for: Local cryotherapy minimally impacts the metabolome and transcriptome of human skeletal muscle
Source: Sci Rep. 2017 May 25;7:2423. doi: 10.1038/s41598-017-02754-5 (PMC5445066; doi:10.1038/s41598-017-02754-5)
Supplement: Supplementary file 1 — Supplementary Table S1 [file 41598_2017_2754_MOESM1_ESM.pdf]

Supplementary Table S1. Primer sequences.

Gene	RefSeq	Forward Primer (5'-3')	Reverse Primer (5'-3')	Amplicon Size (bp)
B2M	NM_004048.2	CACTGAATTCACCCCCACTGA	CTGCTTACATGTCTCGATCCCA	104
CIRBP	NM_001280.2	GTTGTGGTGCGCTGTCTTC	CATGGCGGCCACTGAGTC	60
CSDE1	NM_001007553.2	CTCGCGAGAGAAGCGAGATT	AAGCAGCAGTTTCAGGTGGT	129
RBM3	NM_006743.4	CATGGCGTGAGCTAATCCCT	AGTTCAGTGCATCTCTGCCC	90
YBX1	NM_004559.4	TTAGCCGCCAAAGGTCCAAT	TGTCAATCTGCCCACCTGAC	89
